# *Schadenfreude* is higher in real-life situations compared to hypothetical scenarios

**DOI:** 10.1371/journal.pone.0205595

**Published:** 2018-10-11

**Authors:** Maria Luz Gonzalez-Gadea, Agustin Ibanez, Mariano Sigman

**Affiliations:** 1 Laboratory of Neuroscience, Torcuato di Tella University, Buenos Aires, Argentina; 2 Institute of Translational and Cognitive Neuroscience (INCYT), INECO Foundation, Favaloro University, Buenos Aires, Argentina; 3 Consejo Nacional de Investigaciones Científicas y Técnicas (CONICET), Buenos Aires, Argentina; 4 Center for Social and Cognitive Neuroscience (CSCN), School of Psychology, Universidad Adolfo Ibanez, Santiago, Chile; 5 Universidad Autónoma del Caribe, Barranquilla, Colombia; 6 Centre of Excellence in Cognition and its Disorders, Australian Research Council (ACR), Sydney, Australia; Coventry University, UNITED KINGDOM

## Abstract

*Schadenfreude* (i.e., the pleasure derived from another’s misfortune) has been widely studied by having participants imagine how they would feel in hypothetical scenarios describing another person’s pain or misfortune. However, research on affective forecasting shows that self-judgments of emotions are inaccurate in hypothetical situations. Here we show a study in which we first presented a hypothetical *schadenfreude* situation and few months later, due to an exceptional circumstance, the situation turned out to happen in reality. This fortuitous circumstance allowed us to compare people’s imagined emotional reactions with their actual feelings. Results showed that *schadenfreude* was higher in the real situation than in the hypothetical one. More importantly, participants used different proxies to predict their emotional reaction: while out-group dislike served as a proxy of *schadenfreude* in both types of scenario, the degree of in-group identification also increased *schadenfreude* in those who had experienced the real event, arguably a mechanism to promote positive self-evaluation. These results highlight the importance of assessing *schadenfreude* in the heat of the moment.

## Introduction

The phenomenon of *schadenfreude* (i.e., the pleasure derived from another’s misfortune [1,2]) has been widely studied by having participants judge their (imagined) feelings in hypothetical scenarios involving other people’s pain or misfortune [2–6]. Typical tasks require imagining one’s own reactions upon learning that someone with higher social status or opposite political views has lost his/her job [6,7], or when reading that your least favorite sport team has been defeated [8–10]. These studies have shown that *schadenfreude* is modulated by the deservingness of the other’s misfortune [11–13], the resentment [14] and envy [2,6,15–17] toward the person or group that failed, and one’s positive self-evaluation [15,16,18].

However, this approach is marked by an inherent caveat: people are imprecise at estimating their future emotional states. Research on affective forecasting [19] has shown that predictions of future (i.e., hypothetical) levels pleasure or displeasure are inaccurate. Although a few studies [7,18,20] have been examined *schadenfreude* after real events, no direct comparisons exist of this emotion in imaginary situations vis-à-vis their real-life manifestation. The present study bridges this gap.

We first examined *schadenfreude* by presenting an hypothetical event about the outcome of a fictitious football match in which a long-standing rival (the out-group team) lost to a relatively less competitive team, due to a clearly unfair last-minute decision of the referee. Six months after the study, the very unlikely scenario presented in our task actually occurred in real life. Profiting from this form of scientific serendipity, we repeated the study a few days after the game with a new group of participants that watched the game live and another group that did not watch the game.

Given that previous studies [21–24] showed that people act more like their so-called ought-self (following principles such as moral ideals), we expected that participants who imagined the game would report lower *schadenfreude* than those who watched the real match.

Of note, *schadenfreude* is modulated by different factors. First, some studies [7,25,26] showed that people’s strength of in-group identification predicts *schadenfreude*. Based on the Social Identity Theory (SIT; [27,28]), these studies suggest that malevolence towards an out-group serves to affirm in-group identity and promote positive evaluation of the self [1,27,28]. Other studies focused on football competition [18,20] reported that pleasure for the out-group’s defeat is highly explained by the degree of dislike towards the out-group team. Leach & Spears [18] have found that *schadenfreude* towards a second party (i.e., a second party team had defeated the in-group and then this second party lost to another team) is explained by positive in-group’s evaluation. These authors also showed that *schadenfreude* toward third parties (i.e., a team that had not defeated the in-group but lost to another team) was better explained by negative stereotyping of the out-group, suggesting more malicious *schadenfreude*.

In the current study we examined whether the degree of in-group identification and the level of out-group dislike would be associated with *schadenfreude*. Following previous studies [18,20], we predicted that *schadenfreude* toward a rival team would positively correlate to the degree of out-group dislike in all groups. We also hypothesized that actual experience of the game (i.e., watching a long-standing rival being eliminated from an international tournament) may increase in-group identification and, so that *schadenfreude* in game-watchers would positively correlate to the degree of in-group identification.

Following previous studies [18,20], we assessed *schadenfreude* by asking participants how much pleasure they felt for the out-group’s defeat. We also wanted to know whether *schadenfreude* would be expressed indirectly as pleasure for the third-party’s victory (i.e., the team that won the match and caused the out-group’s defeat). We expected direct and indirect measures of *schadenfreude* to be correlated. We also examined if the degree of out-group dislike and in-group identification would be show a stronger association with the direct *schadenfreude* compared to indirect *schadenfreude*.

## Materials and methods

### Participants

For the first part of the study, we calculated the minimum number of participants (*n* = 46) for a correlational study (power = .80) with a medium effect size (*r* = 0.40). The hypothetical match group comprised 59 Argentinean participants (30 women), ranging between 20 and 60 years old (*M* = 31.15, *SD* = 10.18). These subjects completed an anonymous on-line survey (sent via Facebook and e-mail) including an hypothetical game circumstance. To diversify the sample we asked participants to send the survey to friends. There were no incentives for the participants to complete the survey. This study was completed during the 9^th^ and 10^th^ of December, 2015. For the second part of the study, during the 2016 edition of the Copa América, 120 Argentinean participants completed the same survey in which the same scenario was presented right after the event occurred in the real match. Participants completed the survey four days after the real match (12^th^ of June, 2016). All responders were recruited by the same procedure as in the first part of the study. To avoid possible confounds of repetition effects, we only called participants who had have not answered the first survey. To this aim, we included a question about whether the participants had completed a survey about preferences in football from our laboratory and excluded those who responded affirmatively. In order to control demographic variables among the groups we excluded six participants that were outside from the age-range of the first sample (hypothetical match group). In our original design we wanted to conform a group of participant who have heard about the game or watched the repetition of the goal. Unfortunately, we only recruited 14 participants for this group and thus we decided to exclude them from data analyses.

The final sample for the second part of the study includes 38 participants (23 women, *M* = 32.52 years-old, *SD* = 10.90) who hand neither watched nor heard about the game (did not watch the match group) and 61 participants (25 women, *M* = 31.52 years-old, *SD* = 9.34) who had watched the match live (watched the match group). No differences were observed between groups in terms of age (*F*_(2, 155)_ = 0.23, *p* = .795), education (*F*_(2, 155)_ = 0.03, *p* = .966), or gender (χ^2^ = 3.66, *p* = .160) (see details in Table 1).

**Table 1:** Means, DS and group comparison in demographics, predictors of s*chadenfreude* and control questions.

	Hypothetical match	Did not watch the match	Watched the match	*p*
Demographics				
Gender (women:men)	30:29	25:36	23:15	n.s
Age	31.15 (10.18)	32.55 (10.90)	31.52 (9.34)	n.s
Education*	4.95 (1.19)	4.89 (1.03)	4.95 (1.13)	n.s
Predictors of *schadenfreude*				
In-group identification	7.27 (2.53)	6.42 (2.40)	7.25 (1.97)	n.s
Out-group dislike	2.71 (2.60)	2.34 (1.98)	2.70 (2.30)	n.s
Control questions				
Remember the "Hand of God goal"? (yes:no)	50:9	51:10	30:8	n.s
Appreciation for Maradona	5.46 (2.94)	5.45 (3.21)	5.69 (2.85)	n.s
Moral judgment about the goal	2.95 (2.65)	3.59 (2.78)	3.59 (2.58)	n.s

* Participants responded to a 7-point scale: 1 = Primary school incomplete, 2 = Primary school completed, 3 = Secondary school completed, 4 = College degree incomplete, 5 = College degree completed, 6 = Master’s degree completed, 7 = Ph. D. completed.

All participants gave written informed consent and the study was reviewed and approved by the ethics committee: “Comité de Ética del Centro de Educación Médica e Investigaciones Clínicas (CEMIC)” qualified by the Department of Health and Human Services (HHS, USA): IRb00001745—IORG 0001315.

### Materials and procedure

Before the real game, the hypothetical match group responded to a short survey that included the following hypothetical football match between Brazil (Argentina’s long-standing rival) and Peru (a relatively less competitive third team): “Brazil and Peru are playing a football match. The match is decisive for the both teams’ chances of advancing to the next round. Brazil will make it to the next round with a draw or a win, and Peru will only classify if they win. The match is tied 0–0 when, close to the end, the referee concedes Peru an illicit goal. With this goal, Peru wins the match and Brazil is eliminated from the playoffs”. We asked participants how much pleasure they felt for the Brazilian team’s defeat (Direct *schadenfreude*, DS) and how much pleasure they felt for Peru’s victory (Indirect *schadenfreude*, IS). Six months after the first study, Brazil and Peru met. Nearly exactly as described in the hypothetical scenario, Brazil just needed a draw and Peru had to win in order to qualify to the next stage of tournament. Unexpectedly, 15 minutes before the end of the match the referee gave an illicit goal to Peru. This goal signaled the qualification of Peru to the next round and the elimination of Brazil from the tournament. This event received substantial media attention (see: https://goo.gl/rPxN7a). Four days after the real match we presented a very similar survey in a new population: “This is a real event that happened few days ago during the Copa América: Brazil and Peru played a match that defined who would advance to the next round and who would be eliminated. Brazil will make it with a draw or a win, and Peru will only classify if they win. The match was tied 0–0 when, 15 minutes before the end, the referee conceded an illicit goal to Peru that was not (was with the hand). With this goal, Peru won the match and Brazil was eliminated from the Copa America”. We included the same questions of the first survey. Furthermore, participants were asked to indicate whether they had (1) watched the game, (2) heard about the game or watched a replay of the goal, or (3) not watch the game.

In both surveys we also included questions to assess relevant predictors of *schadenfreude*, as reported in previous studies [18,20]. We asked how much participants were identified with the Argentinean national team (in-group identification) and their degree of “loathing” for the Brazilian national team (out-group dislike). Responses were given in a 10-point scale (1 = *not at all*; 10 = *very much*). We observed no significant differences among groups in these variables (in-group identification: χ^2^(2) = 4.24, *p* = .120; out-group dislike: χ^2^(2) = 4.54, *p* = .79; see details in Table 1) suggesting that the three groups were similar regarding alternative explanations of *schadenfreude*.

In the last part of the survey we included two control questions to test whether participants differed in their dispositional tendency to like unfair goals. To this end, we used a famous illicit goal scored by Diego Maradona (a famous Argentinean football player) against England in the quarter-finals of FIFA’s 1986 World Cup. This goal is known worldwide as “the Hand of God”. First, we asked whether participants remembered this goal. In each group, more than 80% of the sample remembered the goal (see details in Table 1). Second, we asked how much participants liked Maradona because of this goal and how morally acceptable his action was. We observed no significant differences in these measures among the three groups (Appreciation for Maradona: χ^2^(2) = .21, *p* = .941, and moral judgment about the unfair goal: χ^2^(2) = 2.78, *p* = .249, see details in Table 1). These results suggest that the three groups were also similar regarding their dispositional tendency to like unfair goals.

### Data analysis

Since the ordinal measures were not normally distributed, we analyzed them via non-parametric statistics. First, Spearman’s Rho test was used to test the association between both *schadenfreude* measures in each group. Second, to contrast differences in *schadenfreude* among the three groups, we used a Kruskal-Wallis test and *U* Mann-Whitney test for pair-wise group comparisons. Third, to test whether *schadenfreude* was partially explained by the degree of out-group dislike and in-group identification, we conducted two separate ordinal regression analyses which included both *schadenfreude* measures as dependent variables and group as a categorical factor. To test whether the relationship between *schadenfreude* and its predictors varied between groups we analyzed the interaction effects between the group factor and each predictor. The α value for all statistical tests was set at .05. Cohen’s d (*d*) was used as a measure of effect size for significant effects. The data underlying the results presented in the study are available from: dx.doi.org/10.17504/protocols.io.tcbeisn.

## Results

First, we found that both *schadenfreude* measures were highly associated in all groups (hypothetical group: *r*_*s*_ = .770, *p* < .001; did not watch the match group: *r*_*s*_ = .806, *p* < .001; and watched the match group: *r*_*s*_ = .809, *p* < .001).

**Fig 1:**
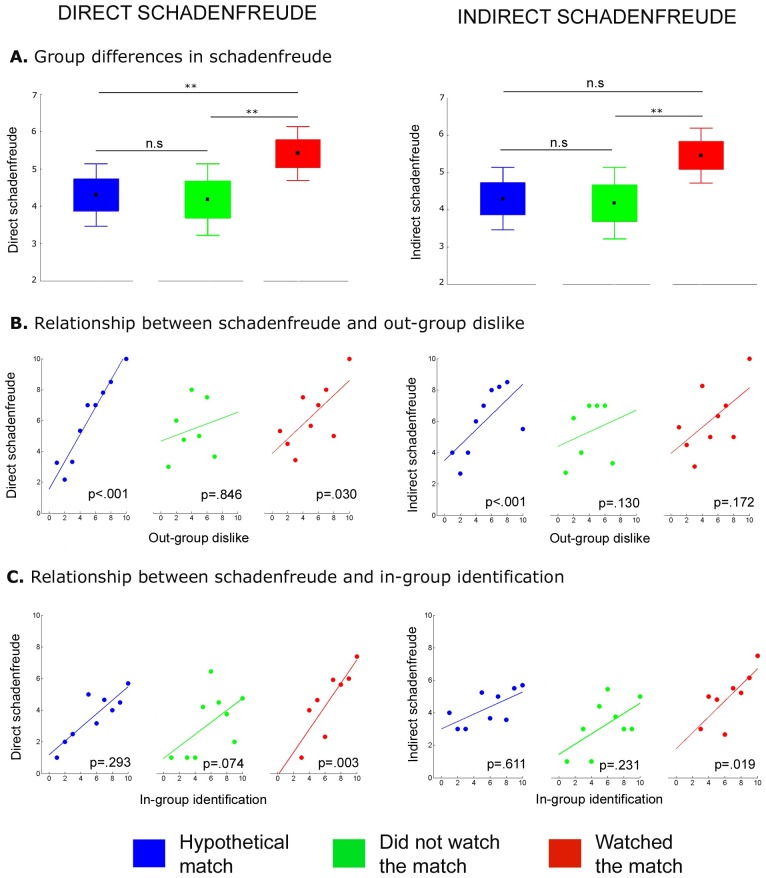
Group comparison in *schadenfreude* and its predictors. **A**. Box plots depict differences in direct and indirect *schadenfreude* between participants who completed the survey in the hypothetical match (blue), those that answered after the real game but did not watch it (green), and the group that watched the match (red). Black points represent the means, boxes the SE, and whiskers ±1.96*SE. Asterisks show significant differences between groups (*p* < .05). **B**. Scatter plots show the association between *schadenfreude* and the degree of out-group dislike in each group. **C**. Scatter plots show the relationship between *schadenfreude* and the degree of in-group identification in each group.

Second, we contrasted differences in *schadenfreude* among the group that watched the match and the two groups that did not experience the situation (those that responded to the hypothetical match and those that did not watch the game; see Fig 1A). As expected, we found significant differences among groups in both *schadenfreude* measures (DS: χ^2^(2) = 6.28, *p* = .043, *d* = 0.33; IS: χ^2^(2) = 6.08, *p* = .048, *d* = 0.33). Participants who watched the match reported significantly higher *schadenfreude* compared to those that did not watch the match in both DS (*U* = 847.00, *p* = .048, *d* = 0.73) and IS measures (*U* = 820.00, *p* = .014, *d* = 1.03). Those that watched the match also reported higher *schadenfreude* compared to those that responded to the hypothetical match. We observed significant differences between these two groups in the DS measure (*U* = 1379.500, *p* = .026, *d* = 0.41) but not in the IS measure (*U* = 1550.00, *p* = .186, *d* = 0.24). As we expected, no significant differences in these measures were found between those that responded to the hypothetical match and the group that did not watch the game (DS: *U* = 1086.00, *p* = .791, *d* = 0.05; IS: *U* = 958.500, *p* = .221, *d* = 0.24). These results suggest that the real experience of the match increased the pleasure for the out-group’s misfortune compared to those that have not experience this event.

Third, we examined whether both *schadenfreude* measures were associated with the degree of the out-group dislike and the level of in-group identification. We observed a significant relationship between DS and the degree of the out-group dislike in the group that watched the game (βE = 1.24, *p* = .030) and the hypothetical match group (βE = 1.66, *p* < .001). However, no significant relationship between these variables was observed in the group that did not watch the game (βE = 1.26, *p* = .846; see Fig 1B left panel). Also, in this model the relationship between DS and the degree of in-group identification was significant only in the group that watched the game live (βE = 1.43, *p* = .003). No significant relationship between these variables was observed in those that had to imagine the match (hypothetical match group: βE = 1.11, *p* = .293, and did not watch the match group: βE = 1.28, *p* = .074; see Fig 1C left panel). The regression model for the IS measure showed that the relationship between this variable and the degree of out-group dislike was only significant for the hypothetical match group (βE = 1.48, *p* < .001). No significant relationship between these variables was observed in the group that watched the game (βE = 1.14, *p* = .172) and those that did not watch the game (βE = 1.27, *p* = .130; see Fig 1B right panel). Lastly, the relationship between IS and the degree of in-group identification was only significant in the group that watched the game (βE = 1.32, *p* = .019), whereas no significant relationship was observed in the other two groups (hypothetical match group: βE = 1.05, *p* = .611, and did not watch the match group: βE = 1.18, *p* = .21; see Fig 1B right panel). Briefly, these results showed that while the degree of out-group dislike was correlated with *schadenfreude* in both hypothetical and real situations, *schadenfreude* was also highly associated with in-group identification in those that had experienced the real event.

Lastly, in the whole sample we found no significant gender differences in any of the dependent and independent measures (see details in Table 2). However, we found that in the group that did not watch the match, women reported significantly lower s*chadenfreude* in the DS measure (*U* = 100.00, *p* = .030, *d* = 0.66), but no significant gender differences were observed in the IS measure (*U* = 115.00, *p* = .089; see details in Table 2).

**Table 2:** Mean, SD and gender differences in dependent and independent measures.

	All participants			Hypothetical match group			Did not watch the game group			Watched the game group		
	Women	Men	p*	Women	Men	p*	Women	Men	p*	Women	Men	p*
Dependent measures												
Direct *schadenfreude*	4.65 (2.93)	4.77 (3.28)	n.s	7.76 (3.06)	3.83 (3.47)	n.s	3.30 (2.51)	5.53 (3.31)	**	5.76 (2.73)	5.22 (3.02)	n.s
Indirect *schadenfreude*	4.72 (2.99)	4.95 (3.10)	n.s	4.93 (2.98)	4.59 (3.47)	n.s	3.26 (2.58)	4.93 (2.56)	n.s	5.80 (2.93)	2.25 (2.98)	n.s
Independent measures												
In-group identification	7.38 (2.26)	6.74 (2.33)	n.s	7.83 (2.16)	6.79 (2.79)	n.s	6.43 (2.45)	6.40 (2.41)	n.s	7.72 (1.99)	6.92 (1.92)	n.s
Out-group dislike	2.65 (2.29)	2.59 (2.39)	n.s	2.87 (2.51)	2.55 (2.73)	n.s	2.22 (1.95)	2.53 (2.06)	n.s	2.80 (2.34)	2.64 (2.29)	n.s

* *U* Mann-Whitney test.

** *p* > .05

## Discussion

Here we capitalized on a highly unique situation—the real occurrence of an extremely unlikely *schadenfreude* event—which allowed us to compare how people thought they would feel in contrast to how they actually reacted when the event happened. The results showed that people underestimated their emotional reaction towards the misfortune of the out-group. More importantly, we showed that this underestimation was not just caused by noise or unreliable estimation of future feelings; rather, these emotional reactions were associated with different proxies. Hence, while the degree of out-group dislike was positively correlated to *schadenfreude* in both hypothetical and real-life scenarios, in-group identification was only positively associated with the level of *schadenfreude* in those who had experienced the match.

Lower *schadenfreude* outcomes in the imagined than the real situation could be partially driven by the subjects’ psychological distance from the event. Previous studies [29,30] have shown that the emotional reactions of situations that are away from the self are often underestimated compared from events that are close. Alternatively, the emotional arousal of the real match might not be elicited during the hypothetical event. This phenomenon, often termed “hot-cold empathy gap”, shows that emotions in the *heat of the moment* tend to be higher than emotions during hypothetical events [24,31–33]. Note that the largest difference between the group that watched the match live and the groups that imagined the match were observed in the DS measure, while differences were smaller for the IS measure. These results suggest that the experience of the match only slightly increased *schadenfreude*. Future studies should replicate these results using a broader assessment of *schadenfreude*, one that includes multiple items for measuring both DS and IS within the research questions.

In this study, we also inquired whether the level of in-group identification and out-group dislike were associated with *schadenfreude*. Leach & Spears [18] found that *schadenfreude* toward a third party was positively associated with stereotypical negative evaluation of the third party. The authors surmised that this malicious felling potentially could escalate into more direct form of derogation and mistreatment to the out-group. Similarly, we found that those with the highest degree of loathing against the out-group reported the highest levels of *schadenfreude* and this correlation was observed in all groups. In addition, we found a significant positive relationship between the degree of in-group identification and *schadenfreude*, but only in those who watched the game. Given that participants first answered to both *schadenfreude* questions and then reported the level of in-group identification, following the SIT [1,25,27], we suggest that the experience of the match may have strengthened the pleasure for the out-group defeat and participants’ identification with the in-group. However, our data do not support any causal link between these two emotions. This possibility should be assessed in futures studies with relevant statistical approaches.

Note that for those that did not watch the game neither the level of out-group dislike nor in-group identification were associated with *schadenfreude*. For this group it was also true that there was a higher percentage of women and that they reported significant lower *schadenfreude* than men in one of the *schadenfreude* measures. On the contrary, previous studies [8,18,20] have reported that gender does not account for individual differences in *schadenfreude* in the domain of football. Nevertheless, these studies have included the level of interest in football as a covariate in their analyses and the authors have argued that this variable appeared to account for the variance that might otherwise be more indirectly explained by gender. Unfortunately, in the current study we did not assessed the participants’ level of interest in football. Future studies should include this measure and test whether it could account differences in *schadenfreude* between real versus hypothetical situations. Additionally, in this study we included only one item to assess each predictor of *schadenfreude*. It would be desirable for futures studies to include more reliable measures to assess these variables.

Here we profited from a naturally occurring event that closely matched the hypothetical situation targeted in our first assessment. This circumstance also introduced problems for the inferences we can make from the data. For instance, it is possible that there were history effects (e.g., differences in the Brazil team’s standing at time of hypothetical vs. real judgments) that could account for differences in schadenfreude rather than the hypothetical vs. real comparison. Although the inclusion of the group of participants that did not watch the game might partially addresses this issue, future studies could strengthened the current approach with experiments that randomly assign participants to the hypothetical and real-life groups.

In short, this study shows that people’s imagined emotional reactions differs with their actual feelings and highlights the importance of assessing moral emotions in the *heat of the moment*.
